# The therapeutic implications of immunosuppressive tumor aerobic glycolysis

**DOI:** 10.1038/s41423-021-00727-3

**Published:** 2021-07-08

**Authors:** Bradley I. Reinfeld, W. Kimryn Rathmell, Tae Kon Kim, Jeffrey C. Rathmell

**Affiliations:** 1 Department of Medicine, Division of Hematology and Oncology, Vanderbilt University Medical Center, Nashville, TN USA; 2 Vanderbilt Center for Immunobiology, Department of Pathology, Microbiology, and Immunology, Vanderbilt University Medical Center, Nashville, TN USA

**Keywords:** cancer, metabolism, immunology, glycolysis, Cancer microenvironment, Cancer metabolism

## Abstract

In 2011, Hanahan and Weinberg added “Deregulating Cellular Energetics” and “Avoiding Immune Destruction” to the six previous hallmarks of cancer. Since this seminal paper, there has been a growing consensus that these new hallmarks are not mutually exclusive but rather interdependent. The following review summarizes how founding genetic events for tumorigenesis ultimately increase tumor cell glycolysis, which not only supports the metabolic demands of malignancy but also provides an immunoprotective niche, promoting malignant cell proliferation, maintenance and progression. The mechanisms by which altered metabolism contributes to immune impairment are multifactorial: (1) the metabolic demands of proliferating tumor cells and activated immune cells are similar, thus creating a situation where immune cells may be in competition for key nutrients; (2) the metabolic byproducts of aerobic glycolysis directly inhibit antitumor immunity while promoting a regulatory immune phenotype; and (3) the gene programs associated with the upregulation of glycolysis also result in the generation of immunosuppressive cytokines and metabolites. From this perspective, we shed light on important considerations for the development of new classes of agents targeting cancer metabolism. These types of therapies can impair tumor growth but also pose a significant risk of stifling antitumor immunity.

The fundamental discovery that led to the field of tumor metabolism was Otto Warburg’s description that tumor tissues utilize glucose and produce lactate in the presence of oxygen [1]. Based on these findings, Warburg proposed a cancer cell-centric model in which disruption of the mitochondrial electron transport chain is necessary for tumorigenesis and thus a commonality among all cancer cells [2]. However, these early studies failed to recognize the duality of metabolic demands by both tumor cells themselves and other resident cells in the tumor microenvironment (TME). Importantly, more recent established literature implicates reprogramming of cell metabolism as essential for immune cell fates. In the context of a tumor, metabolic networks are crucial for immune cell-mediated tumor elimination [3].

It is now clear that dividing cells upregulate glucose metabolism to meet the biosynthetic demands of proliferation [4]. Even though glycolysis produces limited ATP, this metabolic program supports the necessary pathways for de novo lipid, nucleotide, and amino acid synthesis with great efficiency. This applies to both proliferating tumor cells with a deregulated cell cycle and immune cells being activated, which undergo rapid transitions from quiescent to proliferative when confronted with appropriate stimuli. Furthermore, the field of immunometabolism has demonstrated that different immune cell subsets implement and require distinctive metabolic programs to accomplish their diverse effector functions, indicating that the metabolism of proliferating cells shares some but not all features [5]. Because both tumors and immune cells implement generally similar metabolic programs, this review will evaluate possible synergistic interactions between cancer metabolism-targeting therapies and cancer-modulating immunotherapies. Inhibitors developed to target cancer metabolism may therefore, counterproductively, hinder immunotherapeutic efficacy.

## Common signaling and mutational events that regulate glycolysis

The structure of metabolic signaling is shared among most mammalian cells. Critically, the phosphoinositide 3 kinase (PI3K)/Akt/mechanistic target of rapamycin complex 1 (mTORC1) pathway plays a key role in inducing anabolic metabolism for cell growth [6–8]. Ultimately, mTORC1 is the key regulator of metabolic signaling events integrating metabolite availability and growth factor signaling. The roles of these pathways have been reviewed extensively in many cell types [7, 9], and mTORC1 promotes anabolic metabolism by stimulating glycolytic flux via glucose transporter mobilization, hexokinase activation, and glucose-dependent synthesis of nucleotides and lipids via phosphorylation of p70S6 kinase (pS6K) and 4EBP1, which lead to increased protein translation [7] and activation of a variety of metabolic transcription factors. Some of the key downstream transcriptional effectors of mTORC1 signaling are the transcriptional regulator of lipid metabolism sterol regulatory element binding protein (SREBP) and the essential glycolytic transcription factors hypoxia inducible factors (HIF1α/HIF2α) and c-Myc. To ensure proliferation in the presence of sufficient metabolic substrates, mTORC1 induces this anabolic program only when adequate intracellular levels of essential amino acids are sensed at its position on the surface of a lysosome [10].

The transcription factors HIF1α, HIF2α, and c-Myc play crucial roles in promoting glycolysis at the transcriptional level. HIF1α and HIF2α are oxygen-dependent transcription factors that bind to HIF response elements throughout the genome to induce a glycolytic gene expression program. HIF1α, together with the transcriptional coactivator p300, promotes the transcription of glycolytic genes, including Glut1, hexokinase 2 (HK2), aldolase, and lactate dehydrogenase (LDH), to cause increased cellular uptake and utilization of glucose. Active HIF1α elevates the vascular endothelial growth factor (VEGF) level, leading to increased cellular oxygenation and metabolic substrate availability through the formation of new blood vessels. Myc proteins heterodimerize with Max and bind to gene promoters containing E boxes to ultimately drive glycolytic gene transcription. Myc is an unusual transcription factor as it acts broadly across the genome and can associate with paused RNA polymerase II to increase transcript elongation [11]. Therefore, Myc can amplify the expression of a diverse set of active genes with easily accessible chromatin. Unsurprisingly, the Myc family of transcription factors can regulate most of the enzymes related to glycolysis given that these proteins are expressed basally in a majority of cell types. Among the many glycolytic genes that Myc regulates, Glut1, PFK, glyceraldehyde 3-phosphate dehydrogenase, phosphoglycerate kinase, enolase, and phosphoglucose isomerase can have their expression levels increased by Myc accumulation [12]. Additionally, many promoters of glycolytic genes were found to contain Myc-Max-bound E-boxes via chromatin immunoprecipitation [13]. Active Myc is also known to promote glutaminolysis, which can maintain mitochondrial anaplerosis [14] in addition to increasing ribosomal and mitochondrial biogenesis [15].

Over 75% of cancers harbor mutations were predicted to result in a glycolytic phenotype [16] when sequencing data from over 2000 patients were reanalyzed. It comes as no surprise, therefore, that altered metabolism and increased glucose uptake are intimately associated with transformation and a central hallmark of cancer [17, 18]. Nevertheless, most measurements of tumor metabolism are conducted with cell lines in vitro or bulk chunks of heterogeneous tumor tissues in vivo. These in vitro and bulk measurements may simplify or miss key aspects of the metabolism of individual cells due to the altered nutrients available in vitro and the diversity of cellular components and spatial heterogeneity in whole tissues. The interplay among cancer cell, stromal, and immune cell metabolism in vivo, therefore, has not been well disentangled, and there are challenges and opportunities in dissecting this interplay to achieve better treatment of heterogeneous tumors.

## Warburg metabolism as an essential feature of infiltrating immune cells

Previous reviews have extensively discussed the steps required to generate antitumor immunity [19] and the conditions needed for efficacious immunotherapy [20]. To achieve proper antigen shedding, antigen presentation, immune cell activation, immune effector function, and ultimately memory generation, the antitumor compartment of tissue-resident dendritic cells (DCs), M1 macrophages, natural killer (NK) cells and T helper 1 (Th1)/cytotoxic T lymphocytes (CTLs) requires complex metabolic reprogramming. The field of immunometabolism now provides a framework to understand the necessary metabolic changes that promote an effective T cell response to cancer and how cancer cells and immune cells may interact in the TME. These studies illustrate the similarities and differences among these diverse cell types and how nutrient limitations and molecular cues in the TME promote immune cell dysfunction and regulatory immune cell subsets and provide a niche for tumor maintenance and proliferation. The same set of principles can be applied to myeloid cell maturation, phagocytosis, NK cell licensing, and DC antigen presentation, in which ultimate immune cell fate and function are inextricably linked to unique metabolic programs that produce targetable vulnerabilities.

## Metabolic diversity underlies divergent T cell phenotypes and functions

**Fig. 1:**
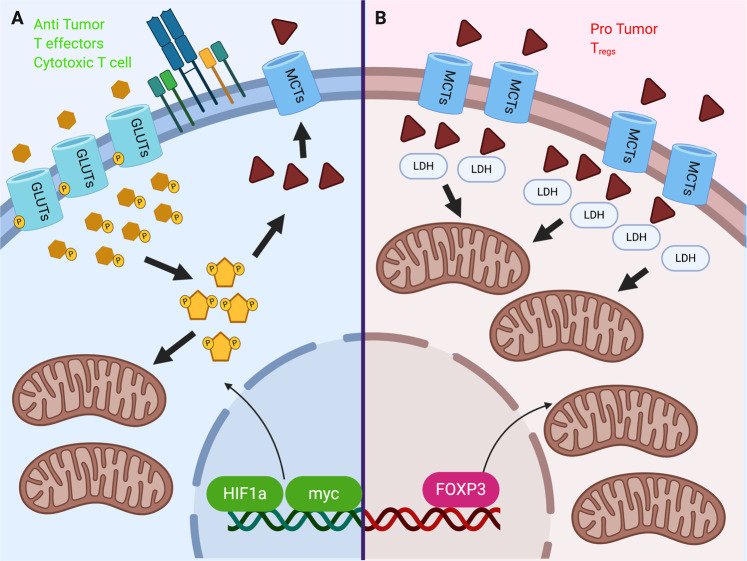
The unique metabolic features of T cell subsets. **A** T cell activation via the TCR results in massive glycolytic reprogramming and a significant increase in mitochondrial metabolism. Recent tracing experiments have demonstrated that glucose is metabolized into both lactate and Krebs cycle intermediates in vivo. These metabolic changes are dictated by the oncogenic transcription factors HIF and myc. **B** Protumor T_regs_ are more oxidative than their antitumor counterparts. These tumor-promoting cells are able to metabolize lactate, convert it into pyruvate via LDH and use this substrate as mitochondrial fuel. The T_reg_-identifying marker FOXP3 drives these substantial increases in mitochondrial biogenesis and function, while mitochondrial complex 3 has recently been shown to be crucial for maximal T_reg_ suppressive function.

Consistent with the understanding of aerobic glycolysis as a program for proliferative metabolism, activated antitumor cells, like transformed tumor cells, employ aerobic glycolysis to perform their functions [3]. T cells must express the master regulator of glycolysis, HIF1α, in addition to the main glucose transporter, Glut1 [21], to perform their antitumor functions. In T cells, the HIF1α and c-Myc protein abundances increase with activation. T cells are unable to proliferate in response to activation with loss of c-Myc but can proliferate with loss of HIF1α [22] (Fig. 1A). However, HIF1α is not dispensable for sustained effector function, as this transcription factor is essential for antitumor immune responses in adoptive cell transfer (ACT) models and immune checkpoint blockade (ICB) [23, 24]. In line with these observations, T cells genetically modified with constitutive HIF activation via loss of the hydroxylation proteins (PHD1/2/3) have an increased glycolytic rate and an increased ability to eliminate lung metastases in a model of metastatic melanoma [25].

Glycolysis is essential in T cell proliferation and activation, and T cell subsets employ different metabolic programs to gain differential effector functions [26]. Th1 and CD8 cytotoxic T cells, for example, are dependent on the uptake of glucose and glutamine through Glut1 and ASCT2 but may be independent of the glutamine-metabolizing enzyme glutaminase (GLS); in contrast, Th17 cells rely on both glutamine uptake and GLS [27]. Conversely, regulatory T cells (Tregs) may be enhanced when glutamine uptake is suppressed. It is important, therefore, to consider how the metabolic constraints of the TME may promote one T cell or myeloid subset over others (Fig. 1B). Additionally, forced expression of the canonical T_reg_ transcription factor FoxP3 decreases active AKT and GLUT1 cell-surface mobilization, illustrating that lineage and metabolic function are intricately linked [28]. Recent data indicate that oxidative metabolism is necessary for T_reg_ function in the TME. Strikingly, mice with T_reg_-specific mitochondrial complex three deficiency have potent antitumor immunity due to loss of the functionality of these suppressive cells [29] (Fig. 1B). This oxidative program includes the uptake of lactate in the TME to sustain suppressive Treg function [30].

## Coreceptor engagement alters metabolism in the TME

**Fig. 2:**
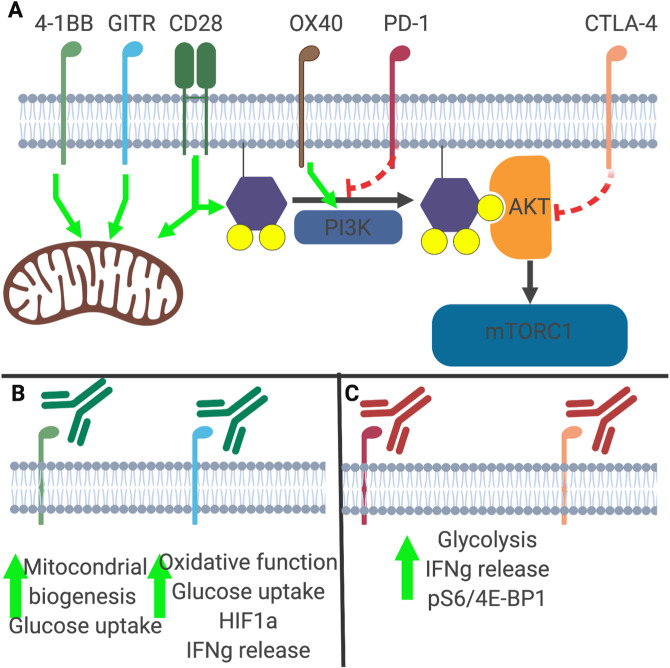
Immunometabolic consequences of checkpoint blockade. **A** Ligation of CD28, 4-1BB, or OX40 promotes increased mitochondrial metabolism and augments PI3K signaling. PD-1 and CTLA4 directly suppress mTOR activation through distinct mechanisms. **B** Using stimulatory antibodies against 4-1BB and OX40 can improve T cell metabolism and enhance tumor elimination. **C** Inhibition of T cell costimulation via blockade of CTLA4 or PD-1 results in increased mTOR signaling. The clinical success of immune checkpoint blockade via PD-1 or CTLA4 inhibition may be due to the ability of these therapeutics to enhance T cell metabolism in tumors.

An often overlooked observation is that the two most clinically relevant checkpoints are negative regulators of T cell glycolysis (Fig. 2). Engagement of the T cell costimulatory receptor CD28 by the ligand B7.1 or B7.2 leads to mobilization of Glut1 and reprogramming for anabolic metabolism via PI3K-AKT and mTORC1. Conversely, CTLA4 both competes for binding with B7.1 and B7.2 and directly recruits the phosphatase SHP2 to inhibit CD28 and TCR signaling and restrict Glut1 translocation, glucose uptake, and T cell activation [31]. Thus, blockade of CTLA4 by ICB removes an inhibitory brake, which results in increased CD28 and TCR signaling and greater levels of T cell aerobic glycolysis. Recent work from Zappasodi et al. demonstrated that CTLA4 inhibition on TME-resident Tregs alters their oxidative program to promote a more glycolytic phenotype. With this shift in metabolism towards increased glucose utilization, Tregs become functionally impaired and thus create a more proinflammatory, antitumor microenvironment [32]. Similar phenotypes have been observed when Tregs are exposed to pathogen-associated molecular patterns, with engagement by immunogenic substrates resulting in increased Treg glycolysis and compromised suppressive activity [33]. This shift is consistent with enhanced PI3K-Akt-mTORC1 signaling, as genetic deletion of the lipid phosphatase PTEN leads to enhanced Akt-mTORC1 signaling that destabilizes Tregs and results in inflammatory autoimmunity [34, 35].

PD-1, another critical T cell immune checkpoint molecule that has been successfully targeted in the clinic, also negatively regulates T cell glycolysis and mitochondrial metabolism [36]. The interaction of PD-1 with PD-1 ligand (PD-L1) blocks glycolysis through inhibition of the PI3K/PKB/mTOR pathway and downregulation of Glut1 [37]. However, PD-1 ligation can also activate AMPK, which triggers fatty acid B-oxidation (FAO) while restraining utilization of branched-chain amino acids [36, 38]. T cell differentiation into effectors requires glycolysis, as described above, in CD28^+^ cells, so PD-1 may block terminal differentiation by inhibiting glycolysis. In contrast, CTLA-4 inhibits glycolysis but not FAO [36]. PD-1 blockade restores T cell glycolysis and IFNγ production in T cells [39]. These two negative regulatory pathways do differ in their mechanism of mTORC suppression (Fig. 2A). PD-1 decreases upstream PI3K activity, whereas CTLA4 increases protein phosphatase 2a and SHP activity to inactive AKT [40]. While the metabolic implications of CTLA4 and PD-1 blockade remain under study, the direct roles related to suppressing anabolic Akt-mTORC1-directed signaling suggests that inhibition is a primary mechanism of action (Fig. 2C).

Unsurprisingly, other T cell inhibitory checkpoints also impact the metabolic fate of tumor-infiltrating T cells (Fig. 2A, B). In line with the suppressive roles of PD-1 and CTLA4 in T cell metabolism, inhibitory coreceptors are now known to decrease the metabolic rate of activated T cells. Lymphocyte activation gene (LAG)-3-deficient naive CD4 + T cells exhibit increased oxygen consumption and enhanced glycolysis via activated STAT5 signaling [41]. The interaction of TIGIT on T cells with CD155 on stomach cancer cells dampens glucose uptake and decreases T cell glycolysis and expression of Glut1 and HK2 [42]. Additionally, TIM3 engagement downregulates glucose uptake and consumption by downregulating Glut1 expression [43]. Stimulation of GITR, a coinhibitory receptor, augments metabolic activities in T cells [44]. Thus, each coreceptor has a distinct function in T cell metabolism.

Activating T cell coreceptors, conversely, can improve the metabolic fitness of activated T cells. 4-1BB agonism activates the liver kinase B1 (LKB1)-AMP-activated protein kinase (AMPK)-acetyl-CoA carboxylase (ACC) signaling pathway, which is important for the metabolism of glucose and fatty acids [45]. Although 4-1BB cosignaling contributes to glycolysis, it induces higher mitochondrial oxidative phosphorylation, leading to the generation of memory T cells rather than the differentiation into effector cells by CD28. 4-1BB signaling also enhances the mitochondrial capacity even in exhausted T cells via p38-MAPK activation [46]. Recent studies have demonstrated that the 4-1BB intracellular signaling domain in chimeric antigen receptor T cells promotes mitochondrial biogenesis and improves oxidative metabolism [47, 48]. In line with the metabolic reprogramming described above, 4-1BB ligation induces Glut1 expression [45]. Stimulation of ICOS, another immunoglobulin superfamily member, enhances glycolysis via activation of mTORC1 and mTORC2 and Glut1 induction [49]. Another TNF receptor superfamily member, OX40, is highly expressed with Glut1 in metabolically active CD4 + T cells [50]. OX40 regulates glycolysis and lipid metabolism in Tregs and promotes T cell expansion and memory T cell differentiation [51]. CD27, normally expressed in resting T cells, provides strong costimulation. CD27 agonism induces the expression of genes involved in glycolysis, glutaminolysis, and fatty acid synthesis [52]. The increased expression of Pim-1 induced by CD27 cosignaling may play a role in glycolysis [53, 54].

Ligands in these T cell checkpoints have metabolic implications on the TME. PD-L1 (also known as B7-H1) has been considered a ligand [55, 56], but it can receive signals as a receptor [57, 58] to impact cancer cell biology independent of the immune system. PD-L1 expression on tumor cells may activate the AKT-mTOR pathway and glycolysis in cancer cells to increase glucose uptake [59]. Interestingly, this type of metabolic reprogramming and resultant microenvironmental acidosis established by lactate secretion combined with hypoxia can upregulate PD-L1 expression via HIF1α and directly lead to inhibition of T cell-mediated cytotoxicity [60, 61]. PD-L1 blockade restores glucose levels in the TME, supporting T cell glycolysis and adequate activation [62]. Among coinhibitory ligands other than PD-L1, B7-H3, also known as CD276, remains to be studied to elucidate its immunological function. B7-H3 interacts with conflicting costimulatory and coinhibitory molecules depending on the context [63]. The nonimmunological roles of B7-H3 include activities in cancer invasion, metastasis, and drug resistance in different cancers [64–68]. Additionally, B7-H3 intrinsically regulates cancer cell metabolism. B7-H3 expression positively regulates HIF1α, leading to glycolysis, lactate production, and tumor growth [69]. B7-H3 also activates the AKT/mTOR pathway, which enhances glycolysis in breast cancers [70], and the STAT3 pathway, which promotes HK2 in colorectal cancers [71]. These findings raise the possibility that coinhibitory ligands such as B7-H3 enhance glucose metabolism in cancer cells, ultimately converting the TME into an overall more suppressive immune environment. Another B7 family member, B7-H4, is a coinhibitory ligand, although its binding partner has not been fully established [72]. B7-H4 on donor or host immune cells prevents graft-versus-host disease (GVHD) lethality in MHC-mismatched bone marrow transplantation models [73]. Genetic deletion of B7-H4 in donor T cells or recipient immune cells enhances mitochondrial activity, superoxide production, Glut1 expression, glucose uptake and metabolism. FAO and fatty acid uptake are also increased in B7-H4^-/-^ T cells in murine GVHD models [73] Whether B7-H4 suppresses glycolysis as well as FAO, unlike PD-1, or inhibits glycolysis rather than FAO remains to be further investigated.

The work above supports a model in which glycolytic metabolism is a component of antitumor T cells and oxidative metabolism is crucial to the T_reg_ suppressive capacity. Adaptation of T cells to the TME, however, can lead to shifts in metabolism and defects in both glycolysis and the mitochondria that directly contribute to impaired immune function. T cells from clear cell renal cell carcinoma (ccRCC) samples were found to have reduced glucose uptake and fragmented and inefficient mitochondria that could be rescued through supplementation with pyruvate or antioxidants or potent costimulation through CD28 [74, 75]. Similarly, T cells from mouse tumors were found to rapidly develop mitochondrial and functional defects, and antitumor immunity could be restored by enhancing mitochondrial biogenesis or promoting lipid uptake to support more efficient mitochondrial metabolism [76]. These data suggest that T cells may adopt multiple metabolic states in the TME and that enhanced rates of aerobic glycolysis may be only one path towards antitumor immunity, with enhanced mitochondrial function being another. The mechanism through which multiple signaling checkpoint ligands are integrated and ultimately alter the metabolic capacity of these crucial cells has yet to be fully elucidated. However, each pathway endows unique signaling and metabolic programs that can impact therapeutic efficacy and patient outcomes. With current technologies, fully dissociating these models remains challenging, as glucose uptake may play roles in supporting both glycolysis and mitochondrial metabolism through pyruvate oxidation. The key distinction yet to be established may be not if T cells utilize glucose metabolism but instead if pyruvate is converted to lactate or provides mitochondrial fuel. Future therapeutic success will be predicated on understanding how tumor-infiltrating lymphocytes (TILs) use metabolic substrates to support their differentiation and antitumor function.

## Tumor cells subvert antitumor immunity via the production of inhibitory metabolites and depletion of essential metabolites in the microenvironment

### Lactic acid and pH as immunosuppressants

**Fig. 3:**
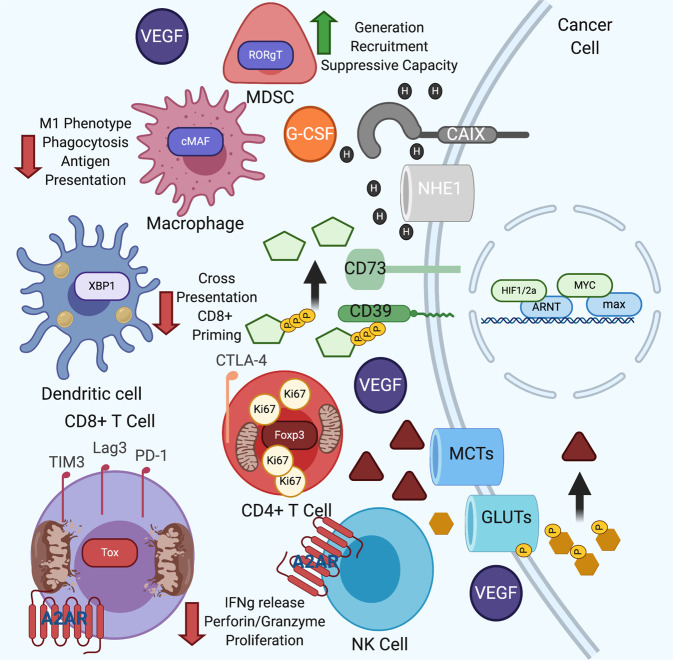
A hostile immunosuppressive tumor microenvironment occurs secondary to tumorigenic mutations. High levels of nuclear myc and HIF increase tumor cell glycolysis, resulting in a TME rich in immunosuppressive molecules. Lactate is produced as a byproduct of oncogene activation. This transcriptional program also decreases the intratumor pH, increases the secretion of suppressive cytokines such as VEGF, recruits suppressive myeloid cells via G-CSF and promotes the extracellular degradation of ATP into adenosine. The combination of these metabolic perturbations and microenvironmental changes decreases the ability of the antitumor immune compartment to perform is requisite functions (seen as fewer cytokines and granzymes in antitumor CD8 + T cells and NK cells). This oxidative microenvironment creates a niche where Tregs, lipid-filled tolerogenic DCs, and suppressive myeloid cells thrive, thus promoting immune evasion and tumor progression.

For aerobic glycolysis to proceed at elevated rates, both tumor cells and immune cells must dispose of intracellular lactate to maintain the cytosolic redox balance and glycolytic flux. Unsurprisingly, the main lactate transporters, MCT1 [77] and MCT4 [78], are transcriptional targets of HIF1α (Fig. 3). With the hypoxic induction of the lactate-generating enzyme LDH [79], the TME is rich in extracellular lactate acid-derived protons. These H^+^ ions are exported into the extracellular space by MCT1, MCT3, MCT4, and the Na^+^/H^+^ symporter NHE1, which is also a HIF1α target [80]. The CO_2_ produced during pyruvate oxidation becomes hydrated extracellularly and transformed into carbonic acid and a free proton via another HIF-responsive gene, carbonic anhydrase IX (CAIX) [81, 82]. Therefore, the TME can be rich in extracellular lactate [74, 83], have a pH as low as 6.0 and be depleted of oxygen. Multiplex immunohistochemistry has confirmed that hypoxic areas in tumors are high in Glut1, LDH, CAIX, and MCT4, demonstrating that these lactate-rich, low-pH environments are truly present in the TME [84]. There is now evidence that these harsh metabolic environments actively evade the immune system. The depletion of oxygen in tumors can have negative consequences for T cell fitness. Hypoxia-experienced CD8 + T cells have compromised mitochondrial metabolism and reactive oxygen species (ROS) tolerance, which prevent tumor clearance [85]. Cancer cell expression of HIF-responsive CAIX can recruit suppressive myeloid cells via expression of G-CSF [86], while melanoma patients who have a high bulk glycolysis transcriptomic signature have relatively poor progression-free survival on PD-1 blockade therapy [87] or adoptive cell therapy [88]. Tumors resistant to combined ICB demonstrate hypermetabolic phenotypes, producing much more lactate in vivo than the corresponding sensitive parental line [89].

Lactic acid is also now recognized as a molecule directly immunosuppressive to all antitumor immune cell types. Human and mouse effector T cells divide less, produce fewer cytokines and are less able to kill cancer cells upon physiologically relevant lactic acid exposure [61, 90, 91]. This inhibition of effector activity is mediated through decreased NFAT translocation into the nucleus secondary to high lactate concentrations [91], a decrease in the intracellular pH [61] and less active p38 and c-JNK/c-JUN [90]. High levels of lactate also promote increased accumulation of Tregs [30, 92], whose presence in the TME promotes tumor progression and metastasis across many tumor types [93]. These tumor-promoting CD4 + T_regs_ appear to use lactate for their oxidative metabolic program, endowing them with a survival benefit in the TME rich in lactate [74, 91, 92, 94]. Loss of the lactate importer MCT1 in a Treg-specific manner was found to result in improved antitumor function, illustrating that this metabolite acts as fuel for these suppressive cells [30]. High levels of lactate are also able to polarize macrophages into a more immunosuppressive M2-like phenotype [95], as demonstrated by expression of Arg1, VEGF, Fizz1, Mgl1 and Mgl2. The mechanism underlying the immunosuppressive action of lactate in myeloid cells is unclear, as the immunomodulatory effects of lactate do not appear to be governed by macrophage expression of GPR81, a G protein-coupled receptor (GPCR) that binds lactate [96]. Lactic acid has been shown to inhibit cytokine production by professional antigen-presenting DCs in organoid coculture models [97]. Similar to other lymphoid-derived immune cells, NK cells cultured in physiologically relevant lactate concentrations show compromised cytokine release [91]. To increase NK cell activity in the TME, tumor-specific knockdown of tumor LDHa has been studied and found to correlate with increased NK cell tumor infiltration and IFNγ + NK cell accumulation. The inhibitory nature of lactate in multiple classes of immune cells may be reminiscent of both PD-1 ligation and CTLA4 ligation in T cells. Lactate may directly decrease the rate of immune cell glycolysis. One study demonstrated that a high extracellular lactate level decreased immune cell glycolysis and limited cellular production of TNFα in macrophages [98]. High levels of extracellular lactate may prevent lactate efflux from infiltrating immune cells to suppress continued flux through the glycolytic program requisite for antitumor function.

Tumor acidity may also be an important modulator of the immune response given that a low intra-lymph node pH regulates T cell proliferation and activation [99]. Ex vivo studies in acidic media have shown that low pH directly inhibits the proliferation of melanoma TILs, limits activation markers such as intracellular p-STAT5 and p-ERK, and restricts the production of IL2, TNFα, and IFNγ. Treatment with proton pump inhibitors led to an increase in the intratumoral pH from 6.5 to 7 and increased the efficacy of ACT [100]. Furthermore, mice drinking bicarbonate ad libitum were found to have a decreased tumor volume with an observed increase in the CD8 + T cell infiltrate. Bicarbonate ultimately improved the efficacy of ACT and ICB in mouse models of melanoma [101]. Modifying the TME pH via inhibition of CAIX also increases response rates to ICB [102]. Given the metabolic complexity of the TME, mouse lymphomas overexpressing the glycolytic/glutaminolytic transcription factor Myc were shown to generate fewer tumor-resident IFNγ+ NK cells. Providing MYC^hi^ mice with exogenous bicarbonate reversed the acidic TME pH and increased NK cell infiltration, NK cell phosphorylation of JNK, and the number of IFNγ-expressing NK cells. In concordance with the increased NK cell activity, mouse survival was increased with excess bicarbonate [103]. These studies suggest that mitigating the acidic environment of tumors, which is induced secondary to increased cellular glycolysis, may improve antitumor immune cell functionality and activity.

### Competition for nutrients

While intratumoral glucose levels may be maintained in some settings [74, 83, 94], metabolic competition for glucose among cells in the TME may contribute to TIL dysfunction in other contexts [59]. Supporting a model in which the increased aerobic glycolysis of cancer cells can restrain TILs, overexpression of PDK1, HK2, Glut1, or c-Myc allowed tumors that were normally rejected to instead grow into palpable masses [59]. T cells purified from those glycolytic tumors had a reduced ability to take up glucose, as assessed with the fluorescent dye 2NBDG [104], and exhibited increased expression of inflammatory cytokines compared with T cells from less glycolytic tumors. Similarly, the nuclear translocation of NFAT, a crucial T cell activation transcription factor, is dependent on the glycolytic intermediate phosphoenolpyruvate (PEP) [105]. In a glucose-limited TME, this necessary event may not occur. Conversely, overexpressing the gluconeogenic enzyme PEPCK1 increases the T cell intracellular PEP level and promotes increased T cell activation and tumor clearance. These findings support a model in which increasing T cell glucose availability may improve tumor eradication and limiting glucose may act as an immunosuppressive mechanism in tumors. It is unclear, however, whether the T cell dysfunction observed in these cases is due to direct metabolic limitations and poor access to nutrients or due to alterations in the immune infiltrate that occur secondarily to a change in cancer cell physiology. It thus is not fully established whether changes to cancer cell metabolism directly alter cancer cell fitness that can then indirectly influence T cell function independent of glucose competition.

Recent work has demonstrated that glucose is present in appreciable concentrations in many mouse models of cancer, as well as human RCC, which supports a model in which glucose is generally not a limiting feature of tumor biology [106]. Using radiolabeled positron emission tomography (PET) tracers, we found that myeloid cells surprisingly consume more glucose per cell than either cancer cells or T cells. Importantly, inhibition of glutamine uptake could further increase glucose uptake, indicating that glucose uptake in the TME is limited by cell-intrinsic metabolic pathways rather than decreasing access to glucose. While microenvironmental glucose limitations may occur, this work questions the widespread nature of glucose limitation and competition in the TME. When nutrients are limiting and competition does occur, this competition may be multifactorial in that there are many diverse cell types attempting to attain and consume metabolic substrates. To overcome such a potential resource barrier when it occurs, immunotherapy may improve T cell competitiveness for glucose uptake or promote alternative pathways, and approaches to increase T cell mitochondrial metabolism have been shown to enhance tumor clearance [74, 75]. However, the glucose availability across tumors may be heterogeneous, and the degree to which glucose competition restricts TILs as a whole remains uncertain, as bulk measurements of glucose in tumor interstitial fluids have found that glucose can be readily available in diverse settings in both mouse tumors and human tumors [74, 75, 83, 94, 106].

While the evidence for glucose competition is mixed, the availability of some nutrients may become limiting for antitumor immune cells in TMEs. There is evidence, for example, that tumors and T cells may compete for the amino acid methionine. For proper T cell activation and cytokine production, methionine must be present [107]. This essential amino acid is crucial for T cell generation of SAM/SAH, which are the key methyl donors in mammalian cells. With decreased methionine uptake, T cells demonstrate an exhausted gene signature and reduced p-STAT5 signaling. This leads to an increase in TME-resident T cell apoptosis, and T cell exhaustion in the TME, which is dependent on tumor cell expression of the methionine transporter, SLC43a2. Intriguingly, in a small trial of human cancer patients, exogenous methionine supplementation significantly improved T cell cytokine production and activation, supporting this model in which antitumor T cells require this amino acid to function [108].

## Glycolytic tumor cells influence antitumor immunity via inhibitory gene networks

### HIF-driven VEGF stimulates the suppressive TME

HIF1α and HIF2α promote not only the expression of glycolytic genes that can lead to lactate accumulation, a reduced pH, and glucose restriction in the TME but also the expression of soluble immunosuppressive factors in the TME. VEGF is considered a canonical HIF target (Fig. 3) [109]. Its induction is thought to promote oxygenation and delivery of vital nutrients to hypoxic tissues via the generation of new blood vessels. However, physiological VEGF concentrations prevent DC-induced T cell activation and promote increased differentiation of suppressive Gr-1+ myeloid-derived suppressor cells (MDSCs) in tumors [110]. VEGF signaling through T cell VEGFR2 restricts T cell proliferation, viability and cytotoxicity [111]. Elevated VEGF expression also promotes high levels of the negative checkpoint molecules PD-1, TIM3, and CTLA4 on tumor-infiltrating T lymphocytes [112]. Myeloid cell VEGF suppresses NK cell activity in the TME [113]. As expected, treating RCC patients with the VEGF receptor inhibitor sunitinib decreases MDSC numbers, promotes the accumulation of IFNγ^+^ T cells and depletes FoxP3^+^ T_regs_ [114]. Given these study results, it is unsurprising that clinical trials combining VEGF inhibitors and ICB are demonstrating an increased response rate compared with those evaluating either therapy alone in multiple disease types [115, 116]. Intriguingly, patients with high T cell and myeloid gene signatures appear to benefit the most from this combination therapy, illustrating that pre-existing immunosuppression may be a predictive biomarker of successful immunotherapy [117].

### Immunosuppressive adenosine generation in the TME is secondary to HIF stabilization

Throughout tumorigenesis, constant cell turnover should be recognized as not merely a process of consumption but also an event that creates a milieu replete with additional metabolites, including ATP and adenosine. Immunostimulatory ATP is released by dying and necrotic cells and can be hydrolyzed to immunosuppressive adenosine by CD39 and CD73 (Fig. 3), which are both ectonucleases and targets of HIF1α [118, 119]. Highly glycolytic tumors convert a majority of the extracellular ATP from apoptotic and necrotic cells into adenosine. This conversion of ATP into extracellular adenosine has several negative consequences for antitumor immunity. ATP itself is a damage-associated molecular pattern (DAMP) that can activate the P2RX7 receptor on tissue-resident CD103 + T cells to promote inflammation and survival of this key cell population via mitochondrial fusion [120]. Engagement of ATP purinergic receptors on DCs increases the vaccination response and cell-surface expression of the costimulatory molecules CD80 and CD86 [121]. Conversely, engagement of the adenosine receptor A2AR is anti-inflammatory and compromises T cell proliferation [122, 123] and T cell cytokine release and increases inhibitory checkpoint molecule (CTLA4 and PD-1) expression [124]. A2AR activation has similar negative effects on NK cell proliferation and activation [125, 126]. Genetic depletion of the receptor A2AR specifically in NK cells was found to increase NK cell proliferation and tissue invasion and ultimately improve tumor elimination in multiple models [125]. In RCC, single-agent A2AR blockade may be successful [127] in part due to HIF stabilization, which is necessary for tumorigenesis in this tumor, thus creating a TME rich in adenosine [128]. Activation of the alternate immunosuppressive adenosine receptor A2BR can also suppress antitumor immunity by increasing MDSC infiltration and maintenance and myeloid VEGF expression [129, 130]. Consistent with an immunosuppressive role for the intratumoral conversion of ATP into adenosine, combining CD73 blockade with ICB results in synergistic inhibition of tumor growth in preclinical models [131].

## Combining metabolic agents with immunotherapy

### Barriers and opportunities for synergy between metabolism-based therapies and ICB

ICB has revolutionized the treatment of many metastatic cancers [132–134]. However, there remains a significant need to enhance the activity of these treatments to drive durable remissions both in more patients and across more disease types. The high rates of resistance to single-agent ICB therapies across all tumor types have led to the development of many trials combining targeted therapies, chemotherapies, or other metabolism-based therapies with ICB in efforts to increase responses [135, 136], yet agents that target cancer metabolism may also impair antitumor immunity. Because in vivo metabolism and heterogeneity can confound in vitro modeling, we propose that using immunocompetent models of cancer will be critical for identifying metabolism- and TME-targeting agents to limit tumor proliferation that simultaneously retain the capability of the immune system to eliminate tumors.

### Warburg metabolism-targeting agents can limit or augment the antitumor response

**Fig. 4:**
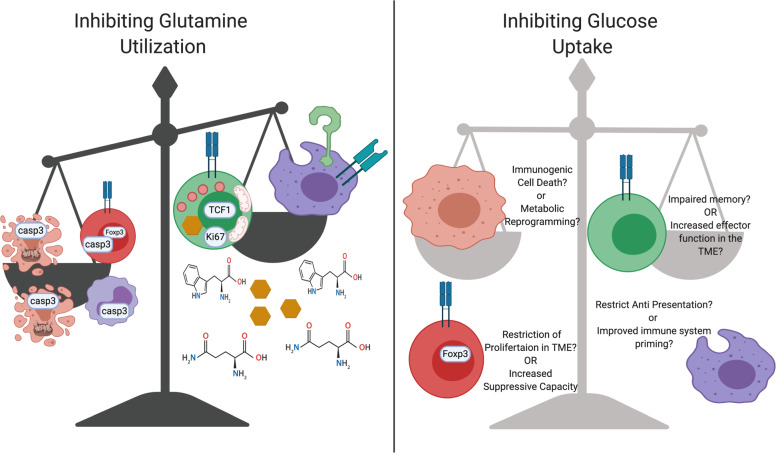
Targeting metabolic pathways may hamper antitumor immunity. Inhibition of glutamine pathways with antagonists, such as DON, or GLS with CB839 promotes antitumor immunity. The TME becomes enriched with glutamine, glucose and tryptophan secondary to these pharmacologic interventions. T cell metabolic reprogramming with glutamine perturbations results in increased expression of antitumor molecules, such as granzyme B and perforin, as well as improvements in mitochondrial function and increased glucose utilization. Glutamine starvation promotes tumor cell death and decreases the functions of MDSCs and Tregs. Antitumor M1-like macrophages increase antigen presentation machinery and inflammatory cytokine production in response to alterations in glutamine metabolism. It is currently unknown how blocking glucose uptake alters immune and tumor cell functions in malignancy. It is possible that this therapeutic targeting of glucose metabolism may restrict antitumor immunity while inducing increased tumor growth. Alternatively, T cell metabolic function may be improved by limiting the metabolic stress these cells experience in the TME. Glucose metabolism is important for macrophage phagocytosis and antigen presentation, and it is currently unknown how restricting glucose aids or inhibits antitumor function.

To support the increased glucose demand of TME-resident cells, glutamine is consumed by both transformed cells and infiltrating cells [3, 137, 138]. As an anaplerotic source to maintain mitochondrial metabolism and amino acid pools and to increase glutathione stores, glutamine metabolism is often coupled to aerobic glycolysis in proliferative cells. Broad inhibition of glutamine metabolism or selective inhibition of GLS/glutamine uptake can result in reduced tumor glycolysis and growth [139–143] (Fig. 4). Importantly, while some T cell subsets rely on GLS, others, including antitumor CD4 + T_h_1 and CD8 + cytotoxic T cells, appear to adapt to glutamine depletion through increased glucose and acetate metabolism. By blocking glutamine metabolism, these antitumor cells can increase effector function [27, 139, 141–143], while tumor cells undergo apoptosis due to overwhelming levels of ROS. It appears that the tumor cell glutamine demand [106] may itself restrict T cell activity in the TME. Deletion of GLS in triple-negative breast cancer was found to result in a marked increase in active TME-infiltrated T cells that acquired excess glutamine via SLC6a14 [143]. Likewise, inhibition of glutamine metabolism in the TME can promote inflammatory M1-phenotype macrophages [144, 145] and impair MDSC infiltration and function via decreased kynurenine generation [146]. In both T cells and macrophages, the mechanism of increased differentiation achieved with inhibition of glutamine appears to be in part mediated through an alteration in the level of the glutamine-derived metabolite α-ketoglutarate, which is required for many demethylation reactions that influence chromatin accessibility and gene expression [27, 144]. A potential drawback, however, may be the induction of terminal differentiation or exhaustion in T cells with inhibition of glutamine metabolism [27], and GLS inhibition can have anti-inflammatory effects in a variety of settings [27, 147] that may also impair antitumor immunity. Combining antagonists of glutamine metabolism with immunotherapeutic agents now has the potential to hinder cancer cell proliferation while promoting inflammatory metabolic programs in T cells and macrophages, although further studies are necessary.

Given that elevated PI3K/mTOR signaling is a commonality among all tumor types, Glut1 is often overexpressed in cancer, and the expression of this transporter is correlated with poor patient outcomes across tumor types [148, 149]. Glut1 inhibition has been shown to be effective in many preclinical models of cancer [150, 151]. However, these studies have been conducted in non-physiological media in in vitro and in xenograft in vivo models that lack an adaptive immune system. Glut1 deficiency may ultimately also prevent antitumor immune cell function (Fig. 4). Effector CD4 + and CD8 + T cells have decreased abilities to proliferate or secrete effector cytokines and promote inflammation when Glut1 is genetically deleted. T_regs_, however, can be Glut1 independent and remain suppressive with Glut1 loss [21]. Additionally, myeloid cell Glut1 loss results in a decrease in the M1-like enzyme iNOS and an increase in the expression of the M2 marker CD206 [152]. CD11c + DCs rely on glucose to differentiate and perform their crucial functions [153]. Careful preclinical evaluation is needed to test whether Glut1 inhibitors compromise antitumor immunity and promote suppressive Tregs and M2-like cells. A therapeutic window may exist where tumor Glut1 levels are relatively high in the tumor compartment when compared to those of immune cells. An appropriate dosing strategy would need to be developed to evaluate an approach where a Glut1 inhibitor could impair tumor growth and metabolism without overly impeding type 1 conventional DC (cDC1) and TIL function. Alternatively, Glut1 treatment may promote long-lived memory T cells with the capacity to provide prolonged tumor control, similar to the effects of 2-deoxyglucose and AKT inhibition in models of ACT [154, 155]. Therefore, future studies should be rigorously conducted to properly evaluate whether Glut1 inhibition in vivo limits T cell glycolysis or instead synergizes with ICB.

### Unique isoform usage creates metabolic vulnerabilities in suppressive immune cells

**Fig. 5:**
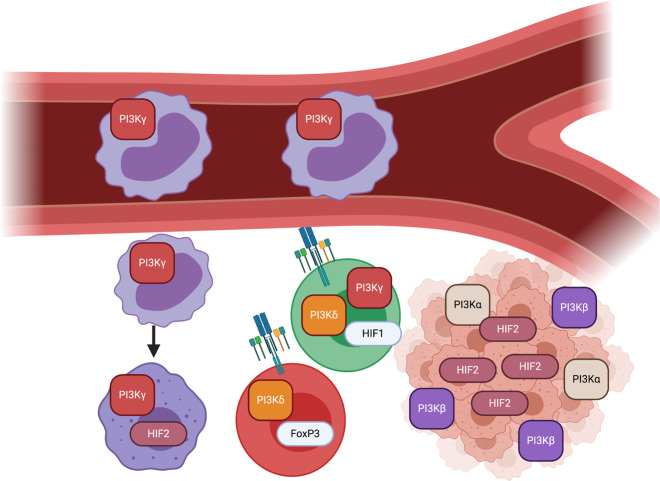
Isoform targeting as a strategy to hamper suppressive immune cell metabolism while enhancing antitumor immunity. PI3kγ is a crucial component in inflammatory myeloid cell recruitment into tumors, MDSC suppressor function, and ultimate lineage commitment to the M2-like macrophage phenotype. HIF2a (in ccRCC and in certain myeloid subsets) is key to sustaining glycolytic function and proliferation. Specific inhibition of PI3Kγ or HIF2a remodels the TME in that there is significant tumor cell death and depletion of CD4 + Tregs and suppressive myeloid cells with enhanced CTL activation and cytokine release.

Whole-genome sequencing of patients with immunodeficiency has led to the discovery that PI3Kγ and PI3Kδ both play important roles in immune cell maintenance. Recent advances in medicinal chemistry now allow specific isoform targeting and thus have provided new insights into methods to augment antitumor immunity without impairing antitumor immune cell glycolysis [156]. The distinct patterns of PI3K isoform usage allows cell-type-specific targeting: malignant epithelial cells express the PI3K isoforms α and β, while myeloid cells express the γ isoform [157] (Fig. 5). In evaluating these PI3Kγ compounds, it has become clear that robust antitumor immunity can be induced by inhibiting glycolytic immature suppressor cells through this unique PI3k variant usage. Myeloid PI3kγ activation is secondary to upstream activation by receptor tyrosine kinases (RTKs), Toll-like receptors (TLRs), and IL1ß [158, 159]. These ligand-binding events mobilize the integrin α1ß4 and release IL10, allowing MDSC tissue infiltration and tumor promotion [158]. Genetic deletion or pharmacological inhibition of PI3Kγ increases the host immune response in both spontaneous tumor models [157, 160] and inflammatory tumor models [161]. Additionally, PI3kγ inhibitors synergize with ICB [162, 163]. Secondary to myeloid cell PI3Kγ loss, there are robust changes in both the infiltrate and cytokines in the TME. By perturbing TME PI3Kγ, increases in infiltrating CD8 + T cells and antitumor conventional DC1s are observed upon depletion of suppressive MDSCs, regulatory B cells and Foxp3+ T_regs_ [164]. MDSCs in PI3kγ-null tumors or inhibitor-treated mice are less able to suppress T cells and less likely to mature into M2-like macrophages [165]. T cells in PI3Kγ-depleted tumors also demonstrate a more active phenotype and a larger antitumor TCR repertoire [162]. Secondary to inhibition of PI3Kγ, the TME becomes enriched with antitumor factors such as IFNγ and IL12 and depleted of immunosuppressive VEGF [166].

The preclinical studies referenced above have led to late-stage clinical trials using PI3Kγ inhibitors in solid tumors in combination with ICB (NCT03961698, NCT03711058, and NCT02637531). However, it is worth noting that excessive PI3Kγ inhibition may ultimately impair the antitumor response. PI3Kγ is known to be expressed in lymphoid cells, such as T and NK cells, as well as DCs [157, 167]. Thymocyte development and mature CD4 + T cells are eliminated in PI3Kγ-knockout (KO) mice [168]. T cells are unable to upregulate the crucial chemokine receptor CXCR3 [169] in the context of PI3Kγ KO [170]. In models of autoimmunity, PI3γ-KO T cells delay graft rejection [171], illustrating that this protein may be responsible for developing T cell responses. Interestingly, ACT of PI3kγ-KO T cells or PI3kγ inhibitor-pretreated T cells generates more memory-like T cells and more robust antitumor responses in multiple cancer models [159, 172]. This work illustrates that antitumor immunity may not rely on T cell PI3Kγ, even though this isoform seems important for de novo T cell generation. Similar to T cells, NK cells with genetic PI3Kγ deletion exhibited impaired IFNγ release [173] and tissue infiltration [174]. PI3kγ loss also impairs cDC1 generation in models of viral immunity, preventing effective CD8 + T cell responses. However, current immunotherapies do not require de novo thymic T cell generation or peripheral DC maturation.

Together, these studies support alterations to the traditional pharmacological approach in oncology. Instead of evaluating metabolic immuno-oncology agents for maximum tolerable doses, the focus should be on developing pharmacodynamic metrics that measure the dose required to achieve the maximal effective immune response to cancer. A recent publication supports this notion in that high-dose (50 mg/kg) PI3kγ/δ inhibition with clinically approved duvelisib (IPI-145) impairs the generation and proliferation of antitumor T lymphocytes. This CTL impairment ultimately counteracts the efficacy of anti-PD-L1 treatment in mouse models of breast cancer. Low-dose treatment (15 mg/kg) with the same compound synergizes with anti-PD-L1 treatment by inhibiting MDSC infiltration and function while promoting the activity of tumor-specific T cells in the TME [175]. The efficacy of this combination may come from the anti-myeloid cell effect of gamma isoform inhibition combined with the anti-Treg feature of low-dose delta isoform inhibition. It is now appreciated that Tregs are uniquely inhibited with PI3kδ inhibition compared to other T cells in mouse and human tumors [176, 177]. These therapeutic windows may exist because of the basal differences in protein isoform usage between immunosuppressive cells and cytotoxic CD8 + T cells noted above.

The differential regulation of glycolysis between tumor and immune cells may also offer an opportunity to selectively suppress tumor glucose metabolism while leaving immune cells intact. Uniquely in ccRCC, HIF2α is able to fully compensate for HIF1α loss [178]. This has led to the development of HIF2α-specific inhibitors for the treatment of ccRCC. Preclinical xenograft models [179, 180] and early-phase clinical trials [181, 182] have demonstrated the efficacy of targeting this transcription factor in vivo and in patients. This is a promising agent for combination with immunotherapy because HIF2α is dispensable for T cell-mediated antitumor immune responses in adoptive cell therapy models [23]. Additionally, myeloid cell-specific deletion of HIF2α decreases tumor infiltration by tumor-associated macrophages in hepatocellular carcinoma and results in decreased tumor cell proliferation [183], so any effect on the immune system may be beneficial. This type of approach would allow antitumor immune cells to upregulate glycolysis via HIF1α without significant impairment.

## Conclusion

In the pursuit of more efficacious cancer therapies, what is becoming increasingly clear is that immune cells in the TME implement discrete metabolic programs to promote tumor elimination or augment tumor progression, offering unique windows for selective therapeutic interventions. A variety of metabolic interventions can preferentially and selectively eliminate tumor cells or subsets of protumor immune cells, providing opportunities for metabolic interventions to serve as strategies to augment checkpoint immunotherapy or, in the future, benefit cellular therapy products. The majority of the pathways that support immune function are well-established pathways, such as the mTOR and PI3k pathways, with increasingly selective agents available for sophisticated tuning of the immune cells in the TME to eradicate tumor cells. The differential dependencies on metabolites, such as glutamine and glucose, are also notable opportunities. Furthermore, it is clear that these strategies offer a sophisticated strategy to harness the immune system, indicating the need for additional immunocompetent animal models to support cancer biology studies. Given these tools and insights, we are poised to make substantive inroads in the treatment of cancer by understanding metabolite consumption patterns in the diverse cells that infiltrate tumors.
